# The Spatial Interaction Effect of Environmental Regulation on Urban Innovation Capacity: Empirical Evidence from China

**DOI:** 10.3390/ijerph18094470

**Published:** 2021-04-22

**Authors:** Ruomeng Zhou, Yunsheng Zhang, Xincai Gao

**Affiliations:** 1 School of Economics, Lanzhou University, Lanzhou 730000, China; zhourm18@lzu.edu.cn (R.Z.); jgygxc@lzu.edu.cn (X.G.); 2 Huamao Financial Research Institute, Henan University of Economics and Law, Zhengzhou 450047, China

**Keywords:** urban innovation capacity, environmental regulation, spatial interaction

## Abstract

This paper applies a spatial econometric model to measure the impact of environmental regulation on urban innovation capacity from a spatial interaction perspective by using panel data from 41 cities in the Yangtze River Delta urban agglomeration from 2009 to 2018. The study findings are as follows: first, environmental regulation has a significant positive impact on urban innovation capacity and a significant positive spatial spillover effect; second, innovation capacity has significant positive spatial dependence; third, city informatization level, government expenditures on science and technology, city economic scale, and industrial development level all positively affect the innovation capacity of neighboring cities and all have positive spatial spillover effects on the innovation capacity of neighboring cities; and finally, city expansion reduces the innovation capacity of a city and has negative spatial spillover effects on the innovation capacity of neighboring cities.

## 1. Introduction

The past 40 years of reform and opening up have enabled China’s economy to achieve world-renowned advances. However, in the wake of this rapid economic development, China now faces a series of ecological and environmental problems, and its past reliance on energy and natural resources as the engines of economic growth is no longer sustainable. The five development concepts of innovation, coordination, green, opening-up and sharing make balancing economic growth with the needs of the ecological environment the theme of China’s development. Meanwhile, China is gradually shifting from high-speed economic development to high-quality development. Environmental regulation can address negative externalities related to environmental problems and market failures and strengthen ecological environmental protections, which is an important measure needed for the government to achieve the goal of balanced environmental and economic development [1]. As one of the means of environmental regulation, the Environmental Protection Law of China was amended in 2014 to solve major environmental problems and guide economic development and environmental protection to develop harmoniously [2,3]. The new environmental protection law requires the State Council to issue a total emission control system for key pollutants, and each province allocates the emission of pollutants to its own cities according to the requirement. Enterprises and public institutions that discharge pollutants are required to pay a pollution fee or an environmental protection tax and to take pollution prevention measures according to the law. Meanwhile, the law requires governments at and above the county level to supervise the implementation of environmental protection laws [4,5].

However, the government’s implementation of environmental regulations means that enterprises must change their production methods and invest additional production resources in green production and emission reduction. This increases costs for enterprises and crowds out their investment in technological innovation. Thus, it hinders the improvement of urban innovation capacity [6]. In recent years, with the introduction of Porter’s hypothesis, some studies have argued that although environmental regulation increases production costs, it may also enable firms to gain compensatory benefits from research and development (R&D) and technological innovation, thereby increasing their competitive advantage. This means that environmental regulation will help to improve urban innovation capacities. These diametrically opposed results have created a gap in theoretical analyses, leading to a rich set of research findings related to environmental regulation and innovation capacity in recent years.

In this regard, researchers have reached three main conclusions regarding the relationship between environmental regulation and innovation capacity. Some argue that environmental regulation significantly promotes urban innovation capacity, while others maintain that environmental regulation hinders urban innovation capacity, and another conclusion also contends that the relationship between the two is uncertain. The key cause of this discrepancy is differences in research perspectives. Studies that focused on the stringency of environmental regulation requirements [7,8], different types of environmental regulation [9,10] and regional heterogeneity [11,12,13] have reached different conclusions. Therefore, the study of environmental regulation and urban innovation capacity has strong practical significance. For China, the in-depth implementation of an innovation-driven development strategy has made innovation an important symbol of the comprehensive competitiveness of cities. Given the current focus on high-quality economic development, exploring the relationship between environmental regulation and urban innovation capacity can help navigate possible conflicts between economic growth and environmental protection.

Although the existing literature offers different research perspectives, studies of the relationship between environmental regulation and urban innovation capacity have ignored the existence of spatial interaction effects between cities. The acceleration of both regional integration processes and urban agglomeration construction has made inter-city connections increasingly close, and environmental regulation may have spillover effects not only on local cities but also on surrounding cities. Thus, geographical relationships warrant attention as an important factor when exploring environmental regulation and urban innovation capacity. Existing studies lack the assessment of the spatial effects of environmental regulation from the neighborhood perspective. Moreover, the studies exploring spatial effects have issues such as overly large scales of geographic units sampled at the provincial level, small sample sizes and insufficient panel data. Based on the findings and shortcomings of existing studies, this paper explores whether environmental regulation effectively stimulates urban innovation capacities from the perspective of spatial interaction. It also discusses the spatial spillover effects of environmental regulation within urban clusters on the surrounding areas.

To study the spatial interaction effect of environmental regulation on urban innovation capacities, this paper selects panel data from 2009 to 2018 for 41 cities in the Yangtze River Delta urban agglomeration and uses a spatial econometric model. The reasons for this approach are as follows. First, as an advanced phenomenon of regional spatial form, the spatial interaction phenomenon is more obvious in city clusters. The Yangtze River Delta city group is the only world-class urban agglomeration in China; thus, it has always been the demonstration area for the development of other regions. China now has 21 national innovation demonstration cities, nine of which belong to the Yangtze River Delta, namely Shanghai, Nanjing, Suzhou, Wuxi, Changzhou, Zhenjiang, Hangzhou, Ningbo and Wenzhou [14,15]. In addition, the Yangtze River Delta became the demonstration zone of green and integrated ecological development in 2019 [16]. As an experiment area of innovation development and green development, other city groups can learn about environmental regulation and urban innovation capacity issues from the Yangtze River Delta. Thus, the study’s results of the Yangtze River Delta will offer good guidance for the development of other city clusters. Second, the provincial data have the modifiable areal unit problem. Therefore, this paper uses city-level data to solve this problem. Furthermore, according to the 2019 Outline of the Integrated Regional Development of the Yangtze River Delta [17], the Yangtze River Delta city cluster has expanded from the original 26 cities to include a total of 41 cities; therefore, this paper considers the expanded cluster with more prefecture-level cities to better reflect the geographic relationship between variables. Finally, since this paper is based on the spatial interaction perspective, it uses a spatial econometric regression model to study the influence of environmental regulation within urban clusters on urban innovation capacities. Compared with the existing literature, this study has two main academic contributions. First, this study enriches the research on the relationship between environmental regulation and urban innovation capacities from the perspective of spatial interaction. Second, this paper uses city-level data to conduct empirical research, which are more reliable than the provincial data commonly used in previous studies.

This paper is organized as follows. Section 2 presents the theoretical hypotheses. Section 3 introduces the study area, selects the research variables and establishes the spatial econometric model. Section 4 analyzes the empirical results and discusses the proposed hypotheses. Finally, Section 5 summarizes this paper’s findings and proposes policy making recommendations.

## 2. Research Hypotheses

Research regarding the relationship between environmental regulation and innovation capacities is rooted in the Porter hypothesis, which suggests that, in an ideal situation, when the government imposes more stringent environmental regulations, enterprises will develop pollution prevention technologies through technological innovation to reduce pollutant emissions as much as possible to meet environmental regulation requirements. It also contends that other innovation agents, such as universities and research institutes, after participating in urban environmental protection research, will also actively invent and innovate to explore clean energy and achieve the protection of natural resources. It posits that with the participation of multiple innovation agents, these kinds of innovative regulations will help boost the technological innovation of enterprises [18]. On the contrary, when the government’s environmental regulation policy is less strict, firms tend to choose negative response strategies and seek to meet the environmental regulation requirements through existing technologies; as government environmental regulations change, firms’ enthusiasm for technological innovation also changes, and due to a lack of sufficient incentives, other innovation agents lose focus on technological innovation. Only when the economy develops to a certain extent are enterprises willing to give up part of their profits for technological innovation, which means that the overall urban innovation capacity has U-shaped characteristics [19,20]. In addition, as an important component of public regulations, environmental regulations are susceptible to interference from phenomena such as rent seeking and corruption, resulting in the failure to implement them and a lack of correlation between urban innovation capacities and environmental regulation [21]. To test whether Porter’s hypothesis is true in the Yangtze River Delta urban agglomeration, this paper proposes the first research hypothesis:

**Hypothesis** **1.** *Environmental regulation can encourage urban innovation agents to participate in technological innovation activities, thus improving urban innovation capacities.*

Since the 18th National Congress of the Communist Party of China, under the strategic requirement of promoting the construction of an ecological civilization, local governments, on the one hand, actively responded to the central government’s call and implemented environmental regulations; on the other hand, it accelerated the promotion of cities’ environmental regulations and enhanced the cities’ innovation capacities due to the push for upgrades by the competition and the effects of environmental regulation from neighboring cities. With the incorporation of location factors into environmental regulation and innovation capacity research frameworks, most research in recent years has focused on the different effects of environmental regulation on regional innovation in different regions. Liu et al. (2020) [22] analyzed China’s provincial panel data and found that in economically developed areas, environmental regulation positively affects technological innovation. Meanwhile, they showed that in economically underdeveloped areas where the government and enterprises are not paying attention to innovation and investment in innovation and budget expenditures are limited, environmental regulations have no obvious effect on innovation. Regarding the spatial spillover effects of environmental regulation on innovation capacities, researchers have not reached a unified conclusion. Zhou et al. (2020) [23] found that although formal environmental regulation has an inverted U-shape relationship with innovation and informal environmental regulation has a positive effect on innovation, they will have an innovation crowding-out effect on industrial enterprises in surrounding areas and produce negative spatial spillover effects. However, He et al. (2020) [24] found that under competing regional environmental regulations, stronger government-based environmental regulations will, instead, stimulate technological innovation in neighboring cities, generating positive spatial spillover effects. In view of these findings, this paper’s second research hypothesis is as follows:

**Hypothesis** **2.** *Environmental regulation has spatial spillover effects on the innovation capacities of neighboring cities.*

Research regarding innovation spillover began with Paul Romer’s endogenous growth theory, which argues that both knowledge and innovation bring positive externalities [25]. According to the first law of geography, in the process of knowledge dissemination, innovations break through city boundaries and spread to neighboring cities. In recent years, among various studies on the spatial spillover effect of innovation, Si (2020) [26] took the cities in the Yellow River Basin as an example, showing that under environmental regulation, green technology innovations can spread to neighboring areas and have positive spatial spillover effects on their innovation capacities. However, due to the profit-seeking nature of both human capital and R&D investment, central regions and economically developed regions will absorb more innovative talents and financial support, which will, instead, inhibit the development of innovation capacities in neighboring regions [27]. By regressing provincial panel data, Li et al. (2019) [28] found that the spatial spillover effects of innovation activities are only significant in the eastern provinces, where innovation spatial networks are more closely connected. Taking these findings into consideration, this paper’s third research hypothesis is as follows:

**Hypothesis** **3.** *Under environmental regulation, urban innovation capacities have spatial spillover effects on neighboring cities’ innovation capacities.*

**Figure 1:**
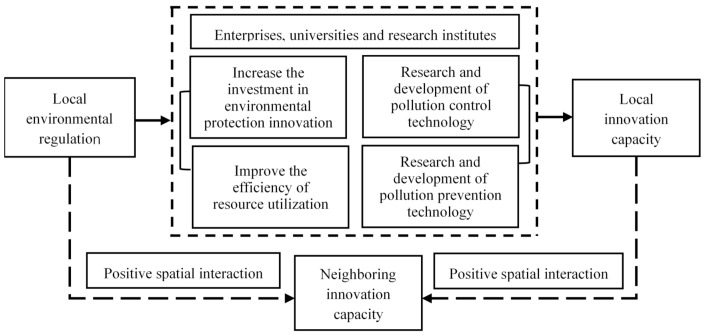
Mechanism of the impact of environmental regulation on urban innovation capacities.

Based on the research hypotheses proposed, this paper elaborates the mechanism of the role of environmental regulation on urban innovation capacities from the perspective of spatial interaction (Figure 1). It argues that environmental regulation will lead the drivers of urban innovation such as enterprises, universities and research institutes to increase innovation investment and seek to improve resource utilization and to develop pollution prevention and control technologies in production processes as well as more environmentally friendly products. These steps can all increase the innovations they produce, thus enhancing local urban innovation capacities. In addition, because of the existence of spatial interactions between cities, local environmental regulations will impact the urban innovation capacities of neighboring places through spatial spillover effects, and local urban innovation capacities will also be linked to and influence the urban innovation capacities of neighboring places through spatial spillover effects.

## 3. Materials and Methods

This section presents an overview of the study area, the selection and treatment of research variables, the selection and design of the empirical model and the data sources. This paper mainly applies a spatial econometric model to empirically test the spatial relationship between environmental regulation and urban innovation capacity.

### 3.1. Study Area

With the rapid progress of urbanization in China, the number and scale of cities are expanding, and the connection between cities is growing closer and closer, as urban agglomerations have become the mainstream and trend of urban development [29]. Moreover, the Yangtze River Delta urban agglomeration is the most dynamic and potential core area in China’s economic development as well as being the only world-class urban agglomeration in China. Studying the relationship between its environment and innovation and development provides an important demonstration and guiding role for the innovation and development of other cities. Therefore, in this paper, the Yangtze River Delta urban agglomeration was selected as the research area to study the impact of environmental regulation on urban innovation capacity.

**Figure 2:**
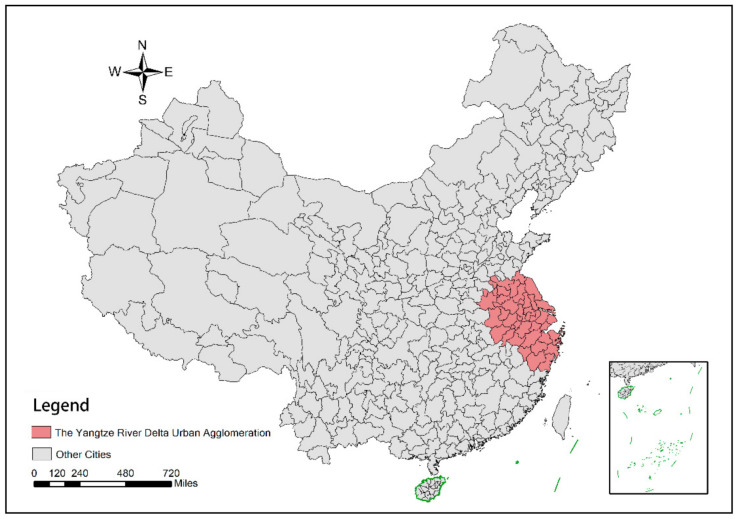
Schematic diagram of the spatial distribution of the Yangtze River Delta urban agglomeration.

The Yangtze River Delta urban agglomeration is located in the eastern part of China, with Shanghai as the central city, and contains 40 prefecture-level cities in Jiangsu, Zhejiang, and Anhui provinces and 1 municipality (A municipality is a city that is governed directly by the central government. China now has four municipalities, namely Beijing, Tianjing, Chongqing and Shanghai.)—Shanghai (Figure 2). As of 2019, the area of the Yangtze River Delta city cluster accounts for 3.7% of China’s land area, the resident population accounts for 16.2% of China’s total population and the total GDP accounts for 23.9% of the national GDP [30], which is an important growth pole of China’s economy. As the intersection of the Yangtze River Economic Belt and the One Belt One Road initiative, the development of the Yangtze River Delta city cluster has had an important radiation and driving effect on the development of the whole eastern region of China.

### 3.2. Variable Selection

#### 3.2.1. Explained Variable

This paper’s explained variable is urban innovation capacity (inno). Innovation generally consists of an innovation stimulus, innovation capacity and innovation performance [31]. Innovation capacity is a reflection of the potential for innovation [32]. Innovation capacity can be proxied by the number of granted patents and the number of invention patents, high-tech exports and expenditures on R&D. We used the number of patent applications granted to represent urban innovation capacity, since granted patents are the result of innovations, which can reflect meaningful innovation activities [33,34,35]. In addition, the number of patent applications granted includes the number of invention patents, utility model patents and design patents from the city’s enterprises, colleges, universities and government institutions, which can well reflect urban innovation capacity.

#### 3.2.2. Explanatory Variable

The explanatory variable in this paper is environmental regulation (regu). Environmental regulation can be classified into command-and-control regulation, market-based regulation and informal regulation [36]. Command-and-control environmental regulation is the government’s compulsory restrictions on industries and residents that involve setting environmental protection laws and regulations. Market-based regulation guides enterprises to reduce pollution by collecting sewage taxes and pollution discharge fees. Informal regulation is the voluntary environmental behavior of the public. In China, command-and-control regulation is the most common regulatory tool, which can be divided into input-oriented regulation and performance-based regulation. The proxies of input-oriented regulation include government environmental subsides, the investment in environmental pollution control, law enforcement frequency of environmental pollution control, the number of employees in environmental protection departments and the number of environmental protection laws and rules issued by the government. The proxy variable of performance-based environmental regulation involves the removal rate of main pollutant contaminants [37]. This paper uses the performance-based environmental regulation composite index to represent environmental regulation [38,39]. Three positive indicators, namely the city sewage treatment rate, the comprehensive utilization rate of industrial solid wastes and the proportion of days with good air quality, are used to construct environmental regulation. The panel data entropy weight method is used to calculate the weight of each indicator [29], and regu is calculated by Equation (5). There are *s*-many years, *n*-many objects and *m*-many indicators. In this paper, *s* = 10, *n* = 41 and *m* = 3. This study first applies the min–max method to normalize the *j*th indicator $X_{itj}$ of object *i* in year *t*, for *t* = 1, …, *s*; *i* = 1, …, *n*; and *j* = 1, …, *m*. The calculation procedures are detailed below.

1. Normalize the positive indicators, the normalized indicator $Z_{itj}$ is:
  (1) $$Z_{itj}=\frac{X_{itj}-\min(X)}{\max(X)-\min(X)}$$
2. Calculate the proportion $P_{itj}$ of indicator *j* from region *i* in year *t*:
  (2) $$P_{itj}=\frac{Z_{itj}}{\sum_{t=1}^{s}\sum_{i=1}^{n}Z_{itj}}$$
3. Calculate the information entropy $E_{j}$ of variable *j*:
  (3) $$E_{j}=-\frac{1}{ln\left(s\times n\right)}\sum_{t=1}^{s}\sum_{i=1}^{n}P_{itj}\times \ln P_{itj}$$
4. Calculate the *j* variable weight $W_{j}$:
  (4) $$W_{j}=\frac{1-E_{j}}{\sum_{i=1}^{m}\left(1-E_{j}\right)}\;$$
5. Calculate the environmental regulation score $regu_{it}$ of city *i* in year *t*:
  (5) $$regu_{it}=\sum_{j=1}^{m}W_{j}\times X_{itj}\;$$

#### 3.2.3. Control Variables

To reduce the estimation bias caused by omitted variables and to effectively control the objective factors that are not considered, the following control variables were selected:

1. The level of urban informatization (info). The informatization level of a city can reflect the ability to exchange and share information of a city. Furthermore, the construction of information exchange platforms and the efficiency of information exchange can ensure that a city introduces innovative resources for re-innovation. This paper used the number of Internet broadband access users to express the informatization level of a city [39].
2. The level of urban science and technology investment (exp). Investment in science and technology can provide financial support for urban innovation activities and reduce the risk of innovation activities due to a long R&D cycle and uncertain R&D results. This paper used the expenditure for science and technology within the general public budget expenditure to express the level of urban science and technology investment [40].
3. City size (size). The size of a city will affect the employment choice of innovative personnel, the location of innovative enterprises and the interaction among innovation entities [23]. We use the urban registered population to represent the city size.
4. The level of urban economic development (ec). The economic level of a city reflects the economic growth rate and growth potential of a city. The economic scale will affect the aggregation of innovative talents and enterprises [41]. This paper used GDP to measure the urban economic development level.
5. The level of urban industrial development (ent). Industrial enterprises constantly carry out technological innovation and invention in the process of daily production and upgrading [41]. In 2015, after the “Industry 4.0” strategic cooperation between China and Germany, to build intelligent industry, industrial companies further became one of the main innovation bodies of cities. Therefore, the number of units of industrial enterprises above a designated size was chosen to represent the level of industrial development.
6. The education level of the urban population (edu). As the incubators of innovation, colleges and universities provide a favorable environment for urban innovation and cultivate potential innovative talents [38]. Therefore, this paper selected the number of students enrolled in regular institutions of higher education to represent the education level of the urban population.

### 3.3. Spatial Econometric Model

#### 3.3.1. Spatial Autocorrelation Test

To investigate whether there is a spatial autocorrelation between inno and regu, this paper tested the environmental regulation index and the global Moran’s I index of the urban innovation ability and environmental regulation of 41 cities in the Yangtze River Delta urban agglomeration from 2009 to 2018. The calculation equation of the global Moran’s I is as follows:

(6) $$\mathrm{Global}\;{\mathrm{Moran}}^{\prime }s\;I=\frac{n\times \sum_{i=1}^{n}\sum_{k=1}^{n}\omega_{ik}\left(x_{i}-\bar{x}\right)\left(x_{k}-\bar{x}\right)}{\sum_{i=1}^{n}\sum_{k=1}^{n}\omega_{ik}\times \sum_{i=1}^{n}{\left(x_{i}-\bar{x}\right)}^{2}}$$

(7) $$\omega_{ik}=\left\{\begin{array}{c} 0\;\left(i=k\right)\\ \frac{1}{d_{ik}^{2}}\;\left(i\neq k\right) \end{array}\right..$$

where *n* is the total number of research cities; *i* and *k* express a certain city, i,k ∈ [1, 41]; $\omega_{ik}$ is the spatial weight calculated by inverse distance to two power weighted method; $d_{ik}$ represents the distance of the latitude and longitude of the two cities; $x_{i}$ is the variable of city *i*; $x_{k}$ is the variable of city *k*; $\bar{x}$ is the mean value of $x_{i}$, where $\bar{x}=\frac{\sum_{i=1}^{1}x_{i}}{n}$; ${\mathrm{Moran}}^{\prime }s\;I$ ∈ [−1, 1], and the closer it is to 1 or −1, the stronger the spatial autocorrelation is.

According to Equation (6), the global Moran’s I of inno and regu were calculated (Table 1). It can be seen that the global Moran’s I index of the explained variable inno is significant at the level of 1%, and they all show the characteristics of positive autocorrelation. The global Moran’s I index of the explanatory variable regu is not significant for the period 2009–2011, but there is a significant positive spatial autocorrelation in 2012–2018. Therefore, both inno and regu have spatial autocorrelation, which indicates that the spatial econometric model is suitable for analyzing the effect of environmental regulation on innovation capacity and their spillover effects. 

#### 3.3.2. Model Selection and Design

After the data passing the Moran’s I test, we assumed the spatial Durbin model (SDM) with spatial and time fixed effects as the research model. To reduce the effects of heteroskedasticity, extreme values and units of measure, this paper’s model was set in logarithmic form [42]. The model is set up as Equation (8).

(8) $$lninno_{it}=\rho Wlninno_{it}+\beta_{1}lnregu_{it}+\beta_{2}Wlnregu_{it}+\overset{6}{\underset{a=1}{\sum }}\gamma_{a}\theta_{it}+\overset{6}{\underset{a=1}{\sum }}\zeta_{a}W\theta_{it}+\varphi_{i}+\tau_{t}+\varepsilon_{it}$$

(9) $$W=\left[\begin{array}{cc} \begin{array}{cc} 0 & \omega_{12}\\ \omega_{21} & 0 \end{array} & \begin{array}{cc} \cdots & \omega_{1n}\\ \cdots & \omega_{2n} \end{array}\\ \begin{array}{cc} \vdots & \vdots \\ \omega_{n1} & \omega_{21} \end{array} & \begin{array}{cc} \ddots & \vdots \\ \cdots & 0 \end{array} \end{array}\right]\;$$

where $inno_{it}$ is urban innovation capacity; *i* denote a city, *i* ∈ [1, 41]; *t* denotes time, *t* ∈ [1, 10]; W is the spatial weight matrix (see Equation (9)); $Wlninno_{it}$ is the spatial lag term of urban innovation capacity, which is used to denote the spatial weighted average of urban innovation capacity; ρ measures the spatial spillover effects of city innovation capacities; $regu_{it}$ represents the environmental regulation index; $\beta_{1}$ measures the contribution of the local environmental regulation to the change in the local city innovation capacities; $\beta_{2}$ measures the contribution of the local environmental regulation to the change in the neighboring cities’ innovation capacities; $\gamma_{a}$ (a ∈ [1, 6]) measures the effect of the city’s control variable *θ* on this city’s innovation capacity; $\zeta_{a}$ measures the effect of the local control variable *θ* on the neighboring cities’ innovation capacity; $\varphi_{i}$ is the regional fixed effect; $\tau_{t}$ is the time fixed effect; $\varepsilon_{it}$ is the random disturbance term.

Following the spatial panel data testing process introduced by Anselin (2006) [43] and Elhorst (2015) [44], we first performed the Lagrange Multiplier statistic test. As Table 2 shows, the estimated values of LM for both the spatial autoregressive model (SAR, also known as the spatial lag model) and the spatial error model (SEM) are significant at the 1% level. Thus, we can reject the original hypothesis that the model does not have spatial lag and spatial error terms. This proves that the spatial correlation between the observations is significant. Next, the results of the robust LM test, *p*_SAR_ = 0.0000 < 0.05 < 0.1840 = *p*_SEM_, indicate that we can accept the original hypothesis that the SDM would not degenerate to the SEM, but we reject the original hypothesis that the SDM would not be reduced to the SAR. Therefore, we chose the SAR to analyze the impact factors of the urban innovation capacity of the Yangtze River Delta city cluster.

The Hausman test for SAR showed a *p*-value less than 0.05 (Table 3); therefore, we chose the fixed effects model. Then, by testing the likelihood ratio of the spatial econometric model (Table 4), we found that the *p*-values of the LR test results for both regional fixed effects and time fixed effects were significant at the 1% level. Therefore, the model should contain spatial fixed effects and time period fixed effects.

Overall, we chose the spatial autoregressive model with the spatial and time fixed effects model to research the spatial effects of environmental regulation on urban innovation capacity; see Equation (10).

(10) $$lninno_{it}=\rho Wlninno_{it}+\beta_{1}lnregu_{it}+\overset{6}{\underset{a=1}{\sum }}\gamma_{a}\theta_{it}+\varphi_{i}+\tau_{t}+\varepsilon_{it}$$

where $inno_{it}$ is urban innovation capacity; *i* denotes a city; *t* denotes time; W is the spatial weight matrix (see Equation (4)); $Wlninno_{it}$ is the spatial autoregressive term of urban innovation capacity; ρ is the spatial spillover effects of $inno_{it}$, measuring the spatial dependence of $inno_{it}$; $regu_{it}$ denotes the environmental regulation; $\beta_{1}$ is the effects of the local $regu_{it}$ on the change in a city’s $inno_{it}$; $\gamma_{a}$ (a ∈ [1, 6]) measures the effect of the city control variable $\theta_{it}$ on a city’s $inno_{it}$; $\varphi_{i}$ is the regional fixed effect; $\tau_{t}$ is the time fixed effect; $\varepsilon_{it}$ is the stochastic disturbance term. Following the spatial panel estimation method developed by Lesage and Pace (2014) [45], this paper used maximum likelihood estimation.

### 3.4. Data Sources

The research sample comprised 41 cities included in the Yangtze River Delta city group. The selected study time interval was from 2009 to 2018. The above data were obtained from the 2010–2019 China Statistical Yearbook [46], the China City Statistical Yearbook [47], the China Urban Construction Statistical Yearbook [48], the China Statistical Yearbook on the Environment [49] and the 2009–2018 Statistical Bulletin on National Economic and Social Development [50]. All data are accessible at the CEInet Statistics Database, CNKI Statistics Yearbook Database and Qianzhan Database. The data for all variables were obtained from municipal districts. Meanwhile, some missing data were smoothed using linear interpolation.

## 4. Results and Discussion

**Figure 3:**
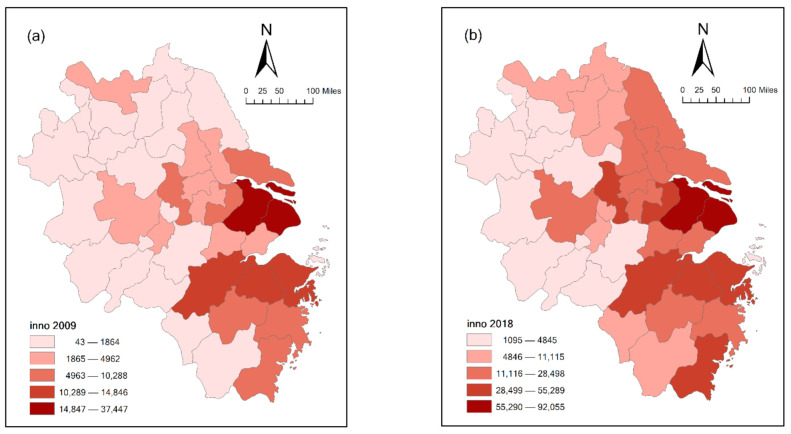
Spatial heterogeneity diagram of urban innovation capacity:(**a**) year 2009, (**b**) year 2018.

**Figure 4:**
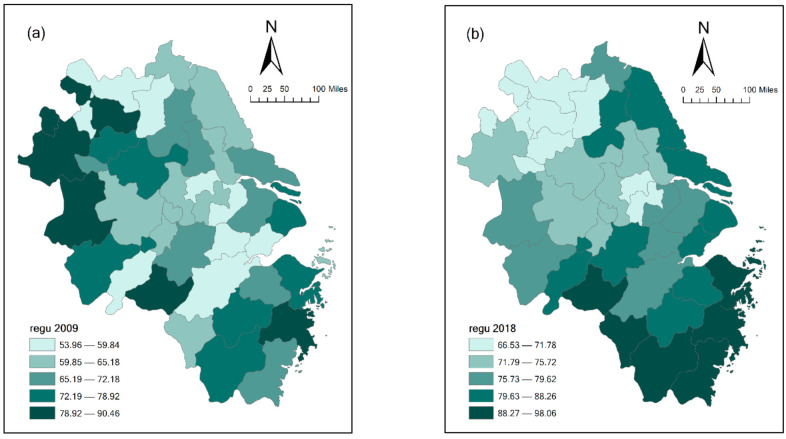
Spatial heterogeneity diagram of environmental regulation:(**a**) year 2009, (**b**) year 2018.

In this section, we first describe the statistical characteristics of the variables in Table 5 and display the spatial characteristics of the main variables in Figure 3 and Figure 4. Then, based on Equation (10), a regression analysis of the factors that affect urban innovation capacity from the perspective of space is performed. The regression results are shown in Table 6, and the results of spatial effect decomposition are shown in Table 7. Table 6 shows that urban innovation capacity has spatial dependence and positive spatial spillover effects. Table 7 shows that a city’s environmental regulation increases its innovation capacity and has positive spillover effects on the neighboring cities’ innovation capacities. To ensure the reliability of the regression results, we tested the robustness of the empirical results, and the test results are shown in Table 8 and Table 9.

### 4.1. Descriptive Statistical Analysis of Variables

Table 5 shows the variables’ descriptive statistics. As shown, except for the level of environmental regulation, the standard deviation of other variables is greater than the mean value, meaning that the data dispersion degree is high, which indicates that the differences in these variables between cities are obvious and the dispersion degree is high. This may be because the construction of an ecological civilization has gradually become the core aim of China’s development strategy since the 18th National Congress of the Communist Party in 2012. Moreover, after the Opinions of the State Council on Accelerating the Construction of Ecological Civilization and the Overall Plan for the Reform of the Ecological Civilization System were issued in 2015, the top-level implementation requirements for environmental regulation have been more clearly defined, and the environmental regulation policy system has been comprehensively enhanced. Therefore, the difference in environmental regulations across the Yangtze River Delta is relatively small.

### 4.2. Spatial Characteristics of the Main Variables

Figure 3a,b shows the spatial heterogeneity of the urban innovation capacities in the Yangtze River Delta city cluster in 2009 and 2018, respectively. The distribution of the innovation capacities of the cities in the cluster shows highs and lows, revealing the characteristics of spatial autocorrelations and spatial spillovers. Several neighboring cities present similar innovation capacity levels. The comparison between 2018 and 2009 indicates that the diffusion effects of the urban innovation capacity of provincial capital cities and the city group’s core city have significantly strengthened, and the radiation effects of urban innovation to neighboring cities have significantly enhanced.

Figure 4a,b shows schematic diagrams of the spatial heterogeneity in the urban environmental regulation index in the Yangtze River Delta city group in 2009 and 2018, respectively. In 2009, the environmental regulation indices for provincial capital cities such as Hefei, Hangzhou and Nanjing were not high; the radiation effect of Shanghai to neighboring cities was not strong; and the spatial autocorrelations were not obvious. However, by 2018, not only were the environmental regulation indices of the cities in the cluster all rising, but distribution also showed obvious highs and lows, and the diffusion effect of provincial capital cities and Shanghai had been significantly strengthened.

Based on these findings, this paper argues that in the Yangtze River Delta region, the innovation capacities of the core cities have relatively obvious radiation effects on the surrounding areas. Moreover, it contends that urban innovation capacities show similar characteristics in geographically adjacent regions, indicating that some spatial spillover among neighboring cities does occur. Meanwhile, as time goes on, the high values of environmental regulation are gradually concentrated around the core cities. This is because after the implementation of the new environmental protection law, the environmental regulation orders of provincial capital cities and core cities in the urban agglomeration were more stringent [5].

### 4.3. Discussion of SAR Regression Results

Based on Equation (10), the regression results are shown in Table 6, which shows that the spatial spillover effects of inno are significantly positive; for every 1% increase in local urban innovation capacity, the innovation capacities of neighboring cities increase by 0.3850%. This indicates that urban innovation capacities have obvious spatial dependence and positive spatial spillover effects among cities in the Yangtze River Delta city cluster and that neighboring cities will have similar levels of innovation capacities. These findings verify Hypothesis 3.

The estimated coefficients of the explanatory variables show that the regression coefficient of regu is significantly positive, indicating that environmental regulation positively impacts cities’ innovation capacities. Meanwhile, the estimated coefficients of the control variables show that the regression coefficient of info is significantly positive, indicating that increased information technology can promote a city’s innovation capacity; the regression coefficient of exp is significantly positive, indicating that investment in science and technology can enhance the local innovation capacity; the regression coefficient of size is significantly negative, indicating that the larger the size of a city is, the lower its urban innovation capacity will be; the regression coefficient of ec is significantly positive, indicating that the higher the economic scale of a city is, the stronger its urban innovation capacity will be; the regression coefficient of ent is significantly positive, indicating that rises in a local city’s industrial level will promote its innovation capacities; and, finally, the regression coefficient of edu is not significant, indicating that the quality of a city’s talent has no significant influence on its innovation capacity.

### 4.4. Discussion of Spatial Effect of SAR

As the spatial econometric model includes spatial interaction effects, the explanatory power of the regression coefficients is not sufficiently accurate. Therefore, direct and indirect effects should be used to explain them. Table 7 reports the direct, indirect and total effects of the explanatory variables on the explained variables.

The direct effect of regu on inno is 1.0677 and is significant at the 1% level, indicating that a 1% increase in a city’s environmental regulation will increase its innovation capacity by 1.0677%. This finding verifies Hypothesis 1. The indirect effect is 0.6076 and is significant at the 1% level, indicating that a 1% increase in a city’s environmental regulation will increase the innovation capacities of neighboring cities by 0.6076%. This finding verifies Hypothesis 2. These results suggest that environmental regulations have encouraged enterprises to eliminate outdated technologies in the Yangtze River Delta city group, that enterprises have received “innovation compensation” by innovating and that environmental regulations can further encourage the innovative activities of urban innovation agents and enhance urban innovation capacities [51]. When the environmental regulation in a city is too strict, enterprises in the city may choose to move to neighboring cities to improve their innovation capacities. Moreover, when a city’s environmental regulation performance is better, the pressure of government competition related to environmental performance will lead neighboring cities to require enterprises to make technological innovations that reduce pollutant emissions [52]. Thus, a city’s environmental regulations will have positive spatial spillover effects on neighboring cities’ innovation capacities.

The decomposition of the effects of each control variable indicates that the direct effect of info is 0.4119 and is significant at the 1% level. This suggests that the urban informatization level significantly promotes a city’s innovation capacity. This is because improvements in informatization levels can enable university teachers and students and enterprises’ R&D departments to obtain more innovative information, data and ideas that can be incorporated into innovation processes and enhance innovation capacities. The indirect effect of info shows that at the 1% level, the innovation capacities of neighboring cities will rise by 0.2330% for every 1% increase in urban informatization level. In addition to enabling the spreading and diffusion of innovation resources more effectively, increases in the level of informatization may also allow industries to spread from central to peripheral areas. This will lead some enterprises in a city to choose to move to industrial parks or specialized small towns in neighboring cities, thereby causing the innovation capacities of neighboring cities to rise [53]. For every 1% increase in a city’s science and technology investment, the innovation capacities of both the city itself and neighboring cities will rise significantly. This is because funds invested in science and technology innovation can promote the accumulation and transformation of a city’s innovation resources. In addition, the Yangtze River Delta city cluster is home to numerous innovation pilot cities, and neighboring governments compete with each other; therefore, the policy of government funding support will have positive spatial spillover effects.

At the 1% significance level, increases in the sizes of both a city and its neighboring cities will reduce the innovation capacity of the city. This is because increases in city size will generate positive externalities at the early stage of urban development, and the population aggregation effect will also attract concentrations of innovative talent and promote the rate of innovation generation. However, when the city’s size exceeds its carrying capacity, continued increases in size will reduce the per capita possession of public resources and reduce the city’s living environment quality and traffic quality. This will make the city gradually less attractive to innovators and hinder improvements to its innovation capacity. Moreover, the expansion of a central city in an urban agglomeration will have a “siphon effect” on neighboring cities, leading to continuous flows of innovative elements such as talent and capital from neighboring cities into the central city [54]. This will cause negative spillover effects on the innovation capacities of neighboring cities. At the 1% significance level, the economic scale of both local and the neighboring cities positively affects the urban innovation capacity, indicating that enhancements in the urban innovation capacities very much need economic support. The direct and indirect effects of ent are both significantly positive, indicating that enhancements in a city’s industrial development level can effectively promote the city’s innovation capacity and have positive spatial spillover effects on the innovation capacities of neighboring cities. This is because the requirement of high-quality economic development leads industrial enterprises to pay more attention to enterprise innovation efficiency, thereby ensuring market competitiveness. This, in turn, promotes the development of high-tech industries and enhances urban innovation capacities. The direct and indirect effects of edu are not significant, indicating that the education level of a city’s residents has no significant effect on the urban innovation capacities. This result indicates that the industrial transformation of universities’ innovation efforts has not gone far enough, and it remains necessary to deepen the cooperation between industry, universities and researchers.

### 4.5. Robustness Test

To test the robustness of the above research results, we first used the method of replacing explanatory variables to re-estimate. The environmental regulation was replaced by input-oriented environmental regulation. We used the environmental subsidy to represent it and selected the expenditure for energy conservation and environmental protection within the general budget of financial expenditure as the proxy indicator. The explained variable and the control variables remained unchanged. Table 8 shows that the regression results of regu are not significant, but the plus–minus signs are still positive. This indicates that the impacts of different types of environmental regulation on urban innovation capacity are different, and the heterogeneity of environmental regulation tools does exist.

To further ensure the robustness and reliability of empirical results, this paper then adopted the method of regressing samples over time intervals to check the model’s robustness. After regressing the samples from 2009 to 2013 and 2014 to 2018, it was found that the direct and indirect effect coefficients of the core explanatory variable regu did not change substantially, and the regression results remained significant (Table 9). This indicates that the original model setting and the regression estimation results are robust.

## 5. Conclusions

Using the 2009–2018 spatial panel data for 41 cities in the Yangtze River Delta city cluster, this paper constructed a spatial lag model to empirically examine the impact of environmental regulation on urban innovation capacities from a spatial interaction perspective. It reached the following conclusions: first, environmental regulation can effectively enhance the innovation capacities of a city and its neighboring cities. Second, urban innovation capacities have positive spatial dependence and can effectively promote the development of innovation capacities in neighboring cities. Third, the level of informatization, government expenditure on science and technology, the scale of a city’s economy and the level of industrial development all have positive effects on the urban innovation capacity of the city and have positive spatial spillover effects on neighboring cities’ innovation capacities. Finally, the expansion of city size reduces the innovation capacity of the city and has negative spatial spillover effects on the innovation capacities of neighboring cities. In summary, the effect of environmental regulation on the enhancement of urban innovation capacities is becoming increasingly significant, and the coordinated development of environmental protection and urban science and technology innovation capacities has, essentially, been realized in the Yangtze River Delta city cluster.

Based on the above findings, this paper puts forward the following policy recommendations. First, within the scope of city clusters, the government should reject concerns that environmental regulation hinders innovation capacity development and, instead, take the path of high-quality development by actively implementing environmental regulation. Doing so will ultimately improve the innovation capacities of local cities and neighboring cities. Second, cities within city clusters should actively implement innovation-driven development strategies, promote the improvement of the innovation capacities of neighboring cities by continuously improving their own innovation capacities and eventually build city clusters into innovation-driven economies. Third, cities undergoing urbanization processes should pay more attention to the optimization of talent structures than mere population size expansion; they should also increase beneficial treatment for the introduction of innovative talent, improve the quality of urban environments and infrastructure construction to make cities more attractive to innovative enterprises and talent and promote innovators’ enthusiasm for innovation. In addition, the precise industry–university–research docking should be improved by, for example, promoting the establishment of joint laboratories between enterprises and universities, optimizing training mechanisms for innovative talent and improving the innovation transformation capacities of other units in cities besides enterprises. Finally, cities should increase investment in technological innovation under environmental regulation, encourage the pursuit of green patents, promote industrial upgrades and the industrial transformation of industries that are not sufficiently environmentally friendly, enhance urban innovation capacities and achieve high-quality economic development.

This study has some limitations that will be solved in future research. First, this paper was not able to study the effect of the different types of environmental regulations on innovation capacity. Second, due to the huge development gap and spatial differences between different regions in China, the study area did not include western and central urban agglomerations. Therefore, future research is needed to focus on the impact of heterogeneous environmental regulations on urban innovation capacities. We will mainly study the different effects of input-oriented regulation (represented by government environmental subsidies), performance-based regulation (represented by the removal rate of main pollutants), market-based regulation (represented by pollution discharge fees) and informal regulation (represented by the number of environmental protection proposals from the public) on urban innovation capacities. Furthermore, from Figure 3, we think that innovation capacity may have significant spatial heterogeneity. In future studies, therefore, it will be necessary to use the geographical detector model to study the determinants of the spatial heterogeneity of innovation capacity. In addition, more regions can be added in our future research. We will study the impact of environmental regulation on urban innovation capacities in different cities. We will consider applying Panel Geographically and Temporally Weighted Regression (PGTWR) in our future research.

## Figures and Tables

**Table 1:** Global Moran’s I index of main variables.

Year	inno	regu
2009	0.5292 *** (4.6427)	0.0116 (0.1128)
2010	0.5371 ***(4.7076)	0.0191 (0.3712)
2011	0.4592 *** (4.0607)	0.0942 (1.0009)
2012	0.4447 *** (3.9482)	0.2351 ** (2.1874)
2013	0.4568 *** (4.0492)	0.3344 *** (3.0060)
2014	0.4844 *** (4.2695)	0.1688 * (1.6500)
2015	0.5175 *** (4.5433)	0.4527 *** (4.0232)
2016	0.4729 *** (4.1721)	0.5594 *** (4.9449)
2017	0.4653 *** (4.1096)	0.6150 *** (5.4108)
2018	0.4731 *** (4.1748)	0.6143 *** (5.3794)

Note: ***,**, and * are significance at 1%, 5% and 10% level, and z-value is in brackets.

**Table 2:** Results of Lagrange Multiplier statistics test.

Null Hypothesis	Statistic	*p*-Value
LM test no spatial	148.6278	0.0000
robust LM test no spatial lag	28.3518	0.0000
LM test no spatial error	122.0444	0.0000
robust LM test no spatial error	1.7684	0.1840

**Table 3:** Hausman test results.

Statistic	Degrees of Freedom	*p*-Value
29.9527	8	0.0002

**Table 4:** Likelihood ratio test results.

Test Statistic	Statistic	Freedom	*p*-Value
LR-test joint significance spatial fixed effects	389.0234	41	0.0000
LR-test joint significance time-period fixed effects	153.3537	10	0.0000

**Table 5:** Descriptive statistics of the variables.

Variables	Mean	Std. Dev.	Min	Max	Obs	Unit
inno	11,922.66	15,762.83	43.00	92,055.00	410	piece
regu	75.99	9.33	53.96	98.06	410	%
info	119.25	123.98	7.00	773.00	410	10^4^ households
exp	163,820.93	463,029.80	1100.00	4,263,655.00	410	10^4^ yuan
size	202.81	222.06	29.00	1462.00	410	10^4^ persons
ec	2214.84	4040.90	112.18	32,679.87	410	10^8^ yuan
ent	1385.75	1992.07	119.00	17,611.00	410	unit
edu	105,628.02	158,315.00	4100.00	859,555.00	410	person

**Table 6:** SAR estimation results of factors influencing urban innovation capacities.

Variables	Estimated Value	*z*-Value	*p*-Value
Wlninno	0.3850 ***	7.4212	0.0000
regu	1.0319 ***	5.2115	0.0000
info	0.3954 ***	3.7453	0.0002
exp	0.0910 **	2.0923	0.0364
size	−0.9753 ***	−5.3036	0.0000
ec	0.6094 ***	3.4905	0.0005
ent	0.3683 ***	3.6455	0.0003
edu	−0.0680	−0.5840	0.5592

Note: *** and ** are significance at 1% and 5% level.

**Table 7:** Decomposition of the SAR spatial effects of variables on urban innovation capacity.

Variables	Direct Effect	Indirect Effect	Total Effect
regu	1.0677 *** (5.2420)	0.6076 *** (3.5788)	1.6753 *** (4.8624)
info	0.4119 *** (3.7483)	0.2330 *** (3.0888)	0.6450 *** (3.6774)
exp	0.0952 ** (2.1072)	0.0541 * (1.8799)	0.1494 ** (2.0631)
size	−1.0113 *** (−5.4148)	−0.5762 *** (−3.5606)	−1.5875 *** (−4.9377)
ec	0.6310 *** (3.4537)	0.3609 *** (2.6873)	0.9919 *** (3.2661)
ent	0.3845 *** (3.8341)	0.2182 *** (3.0646)	0.6027 *** (3.7173)
edu	−0.0743 (−0.6055)	−0.0428 (−0.5979)	−0.1171 (−0.6066)

Note: ***,**, and * are significance at 1%, 5% and 10% level, and z-value is in brackets.

**Table 8:** First robustness test results.

Variables	Direct Effect	Indirect Effect	Total Effect
regu	0.0176 (0.7855)	0.0102 (0.7566)	0.0278 (0.7803)
info	0.4585 *** (4.2142)	0.2664 *** (3.4425)	0.7249 *** (4.1840)
exp	0.0963 ** (2.1606)	0.0563 * (1.9169)	0.1526 ** (2.1065)
size	−0.7789 *** (−4.1425)	−0.4575 *** (−3.0444)	−1.2364 *** (−3.8730)
ec	0.6863 *** (3.6500)	0.4039 *** (2.7754)	1.0901 *** (3.4291)
ent	0.1341 (1.6016)	0.0783 (1.5064)	0.2124 (1.5894)
edu	−0.1030 (−0.8003)	−0.0606 (−0.7818)	−0.1636 (−0.7996)

Note: ***,**, and * are significance at 1%, 5% and 10% level, and z-value is in brackets.

**Table 9:** Second robustness test results.

Year	2009–2013			2014–2018		
Variable	Direct Effect	Indirect Effect	Total Effect	Direct Effect	Indirect Effect	Total Effect
regu	1.6247 *** (4.4674)	0.6445 ** (2.2812)	2.2691 *** (3.9570)	0.5120 ** (2.5980)	0.3839 ** (2.1011)	0.8958 ** (2.4881)
info	0.7440 *** (4.2270)	0.2930 ** (2.3064)	1.0370 *** (3.8732)	0.0772 (0.8417)	0.0569 (0.7932)	0.1340 (0.8321)
exp	0.2511 ** (2.5467)	0.0986 * (1.8396)	0.3497 ** (2.4616)	0.1094 ** (2.6649)	0.0830 ** (2.0734)	0.1924 ** (2.4993)
size	−0.7785 ** (−2.4660)	−0.3033 * (−1.8887)	−1.0818 ** (−2.4274)	−0.6285 ** (−2.3180)	−0.4730 ** (−1.9162)	−1.1015 ** (−2.2240)
ec	0.5842 ** (2.1505)	0.2318 (1.6153)	0.8160 ** (2.0750)	0.3492 (1.5684)	0.2536 (1.4947)	0.6028 (1.5789)
ent	0.1436 (0.8869)	0.0538 (0.7840)	0.1975 (0.8722)	0.2707 * (1.8502)	0.2062 (1.5608)	0.4769 * (1.7660)
edu	0.0672 (0.2908)	0.0241 (0.2442)	0.0912 (0.2801)	0.0395 (0.3343)	0.0316 (0.3408)	0.0711 (0.3403)

Note: ***,**, and * are significance at 1%, 5% and 10% level, and z-value is in brackets.

## Data Availability

The data and code used in this study are available on request from the first author and corresponding author.
